# Ribosomal protein L35 negatively regulates FMDV replication by recruiting AMFR to promote the ubiquitination and degradation of VP2

**DOI:** 10.1128/jvi.01453-25

**Published:** 2025-10-09

**Authors:** Wenhua Shao, Wei Zhang, Yang Yang, Xiaoyi Zhao, Weijun Cao, Chuangwei Chen, Wei Wang, Mengyao Huang, Tingting Zhou, Zixiang Zhu, Fan Yang, Haixue Zheng

**Affiliations:** 1 State Key Laboratory for Animal Disease Control and Prevention, College of Veterinary Medicine, Lanzhou University, Lanzhou Veterinary Research Institute, Chinese Academy of Agricultural Sciences 111658 , Lanzhou, China; 2 Gansu Province Research Center for Basic Disciplines of Pathogen Biology, Lanzhou, China; St Jude Children's Research Hospital, Memphis, Tennessee, USA

**Keywords:** FMDV, VP2, RPL35, ubiquitination

## Abstract

The control of foot-and-mouth disease virus (FMDV) primarily relies on vaccine immunization; however, this approach is not always fully effective, underscoring the urgent need for novel antiviral strategies. This study identifies RPL35 as a host antiviral protein that targets FMDV. Further mechanistic investigations demonstrate that RPL35 directly interacts with the FMDV structural protein VP2, mediating its K48-linked polyubiquitination and subsequent degradation. The Lys217 residue of VP2 is critical for RPL35’s antiviral activity, as evidenced by the increased viral virulence observed with the rO-VP2K217R mutant virus. Through an unbiased proteomic screen, we revealed that RPL35 recruits the E3 ligase AMFR to ubiquitinate and degrade VP2. Additionally, FMDV induces the degradation of KPNA3, thereby blocking RPL35’s nuclear translocation. This study advances our understanding of host-virus interactions and provides new insights into developing antiviral drugs targeting the ubiquitin-proteasome pathway.

**IMPORTANCE:**

This investigation elucidated the antiviral role of RPL35 in the context of FMDV infection. Our results indicate that RPL35 facilitates the recruitment of AMFR, which, in turn, promotes K48-linked polyubiquitination and subsequent proteasomal degradation of the viral protein VP2. This process thereby mitigates viral infection. Further analysis identified Lys217 of VP2 as a critical ubiquitination site for RPL35, with the inhibitory effect of RPL35 being abolished in the recombinant mutant virus rO-VP2K217R. Additionally, we found that FMDV induces the degradation of KPNA3, which obstructs the nuclear translocation of RPL35. Collectively, these findings suggest that RPL35 functions as a potent antiviral effector in suppressing FMDV infection.

## INTRODUCTION

Foot-and-mouth disease virus (FMDV) is a highly contagious pathogen that primarily infects cloven-hoofed animals, such as cattle, pigs, and sheep, causing significant economic losses in the global livestock industry (1, 2). Current prevention and control strategies rely heavily on vaccination; however, the virus’s high genetic variability and the limitations of existing vaccines underscore the urgent need for novel approaches (3, 4). The FMDV capsid, composed of the structural proteins VP1, VP2, VP3, and VP4, plays a critical role in viral assembly, stability, and infection processes (5–7). Among these proteins, VP2 is a key structural component that significantly contributes to the viral lifecycle, making it a focal point for both scientific investigation and applied research (8).

The VP2 protein is crucial for FMDV’s ability to infect host cells, maintain viral particle stability, and potentially evade immune responses (9). Investigating VP2 functions offers valuable insights into virus-host interactions and the mechanisms underlying viral pathogenesis. Moreover, VP2 contain key antigenic epitopes capable of eliciting immune responses, thereby providing a foundation for developing more effective and broad-spectrum FMDV vaccines (10). Beyond its role in vaccine development, VP2 also shows promise as a target for antiviral drug discovery. By elucidating its structural and functional roles in viral replication and assembly, researchers may identify potential targets for small-molecule inhibitors or other therapeutic interventions (11). Disrupting VP2 function could effectively suppress viral replication and transmission, presenting a novel strategy for controlling FMDV outbreaks (12, 13).

Ribosomal proteins (RPs), traditionally recognized for their roles in protein synthesis, have recently been identified as significant contributors to viral infections (14–16). RPL35, a component of the ribosomal subunit and a member of the RP family, has been implicated in various stages of the viral life cycle. Ribosomes, essential for cellular protein synthesis, consist of large and small subunits and are frequently exploited by viruses through mechanisms such as internal ribosome entry sites (IRES), which facilitate viral protein synthesis (17, 18). Interactions between viral proteins and RPs are well documented. For example, RPSA interacts with FMDV VP1 to enhance viral replication (19); RPL4 modulates viral replication as an interaction partner of IBDV VP3 (20), and RPL18 is involved in respiratory syncytial virus (RSV) infection by binding to the nucleocapsid protein (21). Similarly, RPL9 aids in virus particle assembly as a binding partner of MMTV Gag (22), and RPL13 promotes IRES-driven translation of FMDV in a helicase DDX3-dependent manner (23).

Although RPs are often associated with promoting viral infections, recent studies have revealed their potential as antiviral agents. Two primary antiviral mechanisms have been proposed: (i) direct interaction with viral proteins to inhibit viral transcription or translation, as demonstrated by RPL9 binding to the phosphoprotein P of the rabies virus (RABV) (24), and RPS10, 18S rRNA, and tRNAs forming a complex with the HIV-1 Nef protein to reduce viral protein synthesis (25); and (ii) activation of antiviral defense signaling pathways, exemplified by RPS20 modulating Toll-like receptor 3 (TLR3) to inhibit classical swine fever virus (CSFV) replication (26), and RRL13a assembling an interferon-γ-independent antiviral complex to suppress the translation of the respiratory syncytial virus matrix protein M (27).

In this study, we identify RPL35 as a potential inhibitor of FMDV by targeting the VP2 protein. Our findings suggest that RPL35-mediated degradation of VP2 could provide a novel therapeutic strategy for treating FMDV infections. This research not only advances our understanding of FMDV biology but also provides a foundation for developing innovative vaccines, antiviral drugs, and control strategies.

## RESULTS

### RPL35 interacts with the FMDV VP2 protein

**Fig 1:**
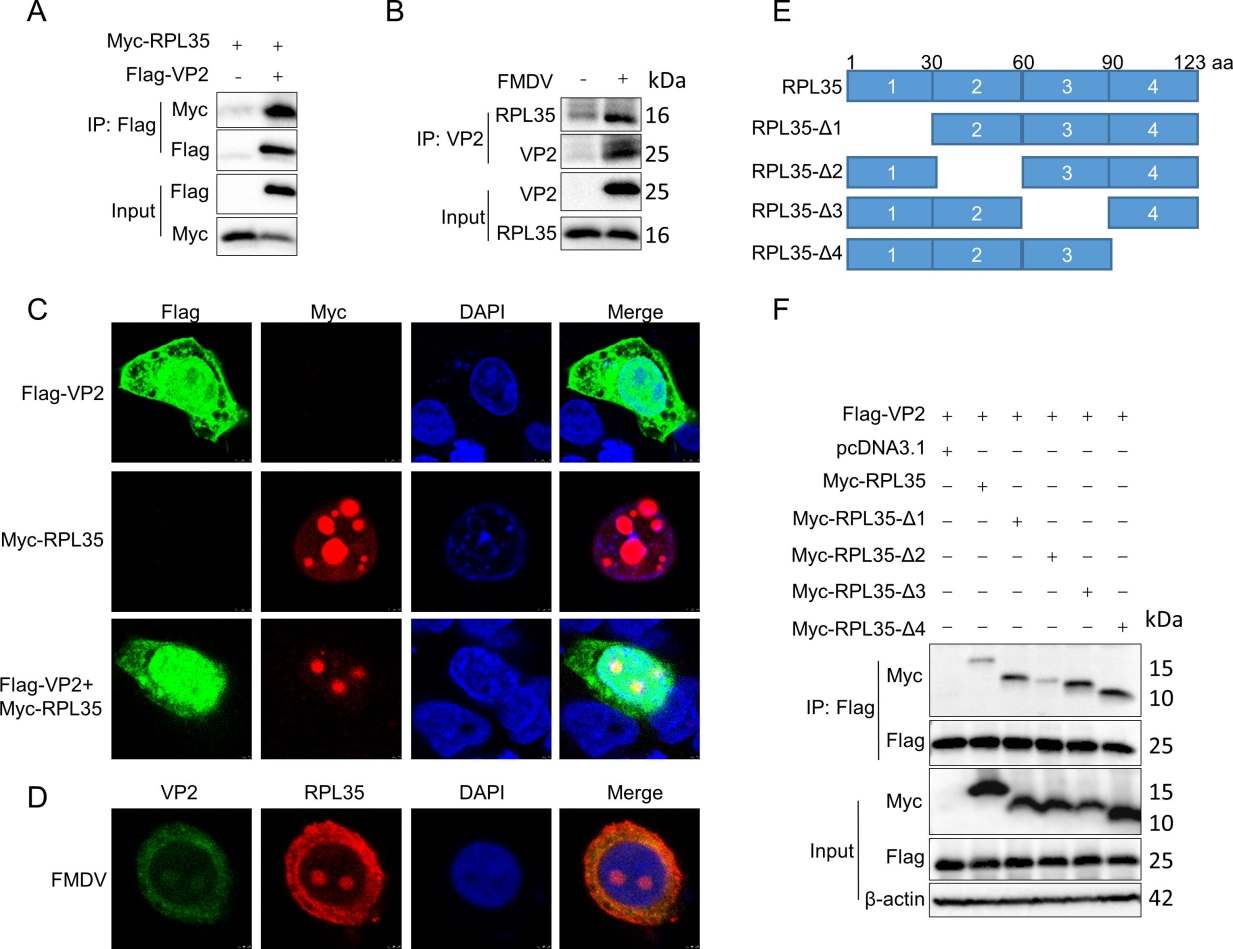
RPL35 interacts with FMDV VP2 protein. (**A**) An exogenous co-immunoprecipitation (Co-IP) assay was performed using HEK293T cells. The cells were transfected with Flag-VP2 and Myc-RPL35 or a control vector. Cell lysates were then subjected to immunoprecipitation using Flag antibodies, followed by immunoblotting with Myc and Flag antibodies. (**B**) The interaction between endogenous RPL35 and VP2 was assessed during FMDV infection. IBRS-2 cells were infected with or without FMDV for 12 h. Subsequently, cell lysates were immunoprecipitated with VP2 antibodies and analyzed for the presence of RPL35 and VP2. (**C**) The colocalization of RPL35 and VP2 was examined. IBRS-2 cells were transfected with Flag-VP2 and Myc-RPL35 for 24 h, then analyzed by immunofluorescence staining using Myc (red), DAPI (blue), and Flag (green) for visualization. (**D**) The colocalization of endogenous RPL35 and VP2 was examined. IBRS-2 cells were infected with FMDV for 8 h and analyzed by immunofluorescence staining using RPL35 (red), DAPI (blue), and VP2 (green). (**E**) A schematic representation of the truncated mutants of RPL35 is provided. (**F**) Western blot analysis of Co-IP assays was performed to delineate the binding domain of the RPL35 protein. HEK293T cells were transfected with Flag-VP2 and Myc-RPL35 or its truncated variants, followed by immunoprecipitation of cell lysates with Flag antibodies and subsequent immunoblotting with Myc and Flag antibodies.

It has been reported that the FMDV VP2 protein plays a critical role in viral replication by contributing to virus assembly, stability, host cell recognition, immunogenicity, and release. To identify host proteins that interact with FMDV VP2, immunoprecipitation coupled with mass spectrometry (IP-MS) was performed. Through an unbiased screening approach, the cellular protein RPL35 was identified as a candidate interacting partner. To validate the interaction between VP2 and RPL35, co-immunoprecipitation (Co-IP) assays were conducted, confirming that RPL35 binds to VP2 (Fig. 1A). To further investigate the endogenous interaction between VP2 and RPL35, cellular lysates from FMDV-infected cells were subjected to immunoprecipitation. The results demonstrated that VP2 interacts with endogenous RPL35 under viral infection conditions (Fig. 1B). Additionally, confocal microscopy analysis revealed that RPL35 colocalizes with VP2 in cells (Fig. 1C), a finding also confirmed in endogenous experiments (Fig. 1D). To map the functional domain of RPL35 responsible for its interaction with VP2, a series of truncation mutants of RPL35 were generated using PCR-based site-directed mutagenesis (Fig. 1E). Co-IP analysis of these mutants indicated that two or more regions of RPL35 are involved in the interaction with VP2 (Fig. 1F). Collectively, these findings provide compelling evidence that RPL35 interacts with the FMDV VP2 protein.

### RPL35 inhibits FMDV replication

**Fig 2:**
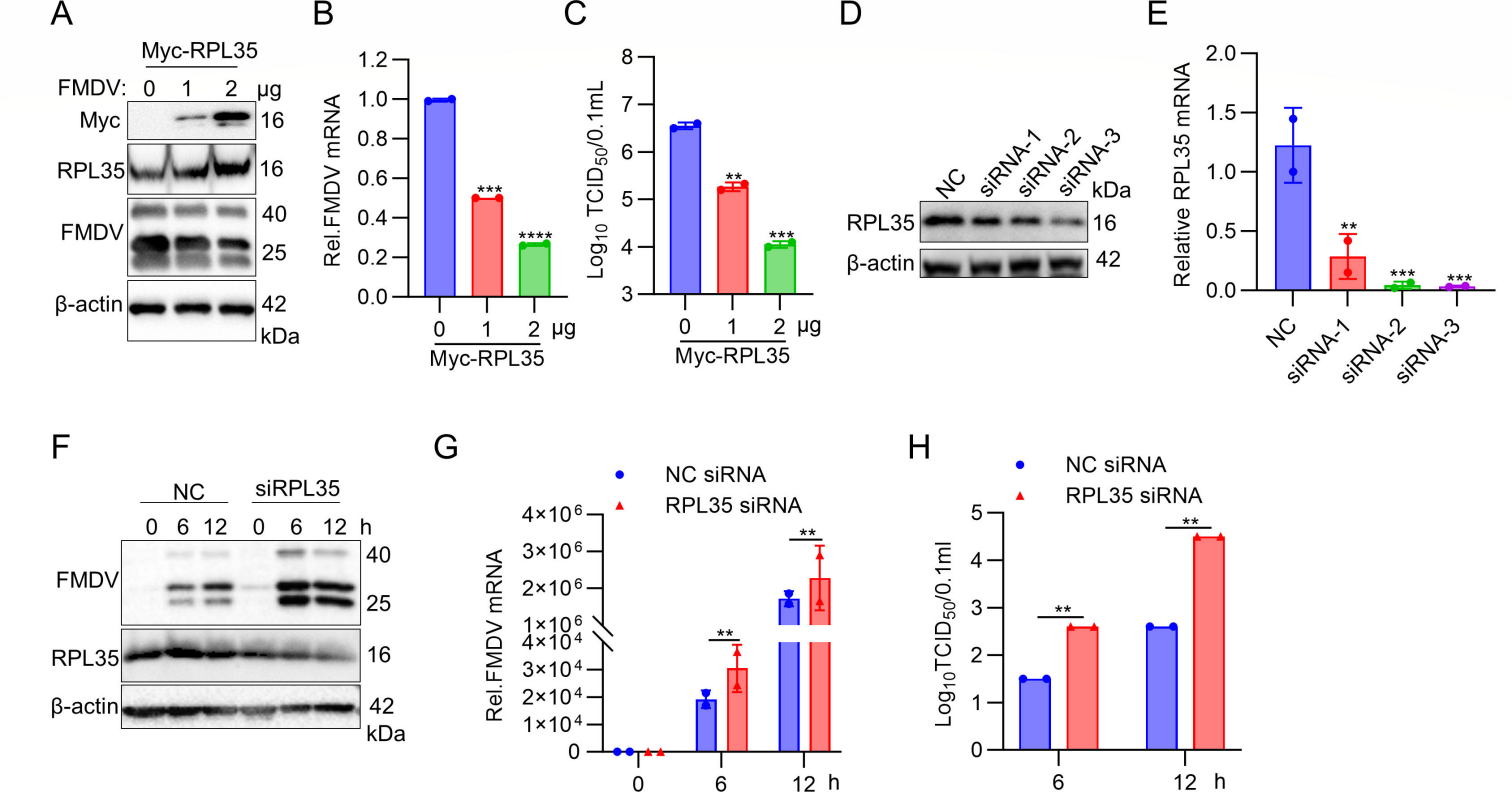
RPL35 inhibits FMDV replication. (**A–C**) The replication of FMDV was inhibited by the overexpression of RPL35. PK-15 cells, seeded in 6-well plates, were transfected with RPL35 plasmids in a dose-dependent manner for 24 h and subsequently inoculated with FMDV at an MOI of 1 for 12 h. The infected cells were harvested to assess RPL35 and FMDV VP2 protein expression using immunoblotting (**A**), to measure FMDV mRNA abundance via RT-PCR (**B**), and to determine viral titers using the TCID_50_ method (**C**). (**D and E**) The efficiency of NC or RPL35 siRNA in silencing RPL35 expression was evaluated. PK-15 cells, seeded in 6-well plates, were transfected with 150 nM of either NC or RPL35 siRNA (si-1, si-2, and si-3) for 36 h. The knockdown efficiency was then assessed by RT-PCR (**D**) and immunoblot analysis (**E**). (**F–H**) The downregulation of RPL35 enhances FMDV replication. PK-15 cells, seeded in 6-well plates, were transfected with 150 nM of NC or siRNA-3 targeting RPL35 for 24 h, followed by infection with FMDV (MOI = 1) for 0, 6, or 12 h. The protein expression levels of endogenous RPL35 and viral VP1 proteins, as well as the viral RNA and FMDV yields, were analyzed using immunoblotting (**F**), RT-PCR (**G**), and the TCID_50_ assay (**H**), respectively. **P < 0.01; ***P < 0.001; ****P < 0.0001.

To investigate the impact of RPL35 on FMDV infection via its interaction with the VP2 protein, PK-15 cells were transfected with Myc-RPL35 and subsequently infected with FMDV. Viral protein expression, viral RNA levels, and viral titers were assessed using immunoblot analysis, RT-PCR, and median tissue culture infective dose (TCID_50_) assays, respectively. Notably, these results revealed that overexpression of RPL35 reduced FMDV protein levels, RNA levels, and viral titers in a dose-dependent manner (Fig. 2A through C). Collectively, these results indicate that RPL35 overexpression significantly suppresses FMDV replication.

To further investigate the role of RPL35 in FMDV replication, three RPL35-specific siRNAs (siRNA-1, siRNA-2, and siRNA-3) were designed and synthesized. Their effects on FMDV replication were assessed in RPL35-knockdown PK-15 cells. The silencing efficiencies of the siRNAs were evaluated using immunoblot analysis, which revealed that siRNA-3 exhibited the highest efficiency in reducing RPL35 expression (Fig. 2D and E). Consequently, siRNA-3 was selected for subsequent RPL35 knockdown experiments. PK-15 cells were transfected with either negative control (NC) siRNA or siRNA-3, followed by FMDV infection. Viral protein levels, viral RNA, and viral titers were compared between siRNA-3-transfected and NC siRNA-transfected cells at specified time points post-infection. Immunoblot analysis demonstrated that RPL35 knockdown significantly enhanced FMDV protein expression (Fig. 2F). Similarly, RT-PCR results indicated a marked increase in FMDV genomic RNA levels in RPL35-knockdown cells (Fig. 2G). Additionally, TCID_50_ assays revealed higher viral titers in RPL35-knockdown cells compared with NC siRNA-transfected cells (Fig. 2H). Collectively, these findings demonstrate that FMDV replication is significantly elevated in RPL35-knockdown cells, underscoring the role of RPL35 in restricting FMDV replication.

### RPL35 affects FMDV RNA synthesis and virus assembly/release

**Fig 3:**
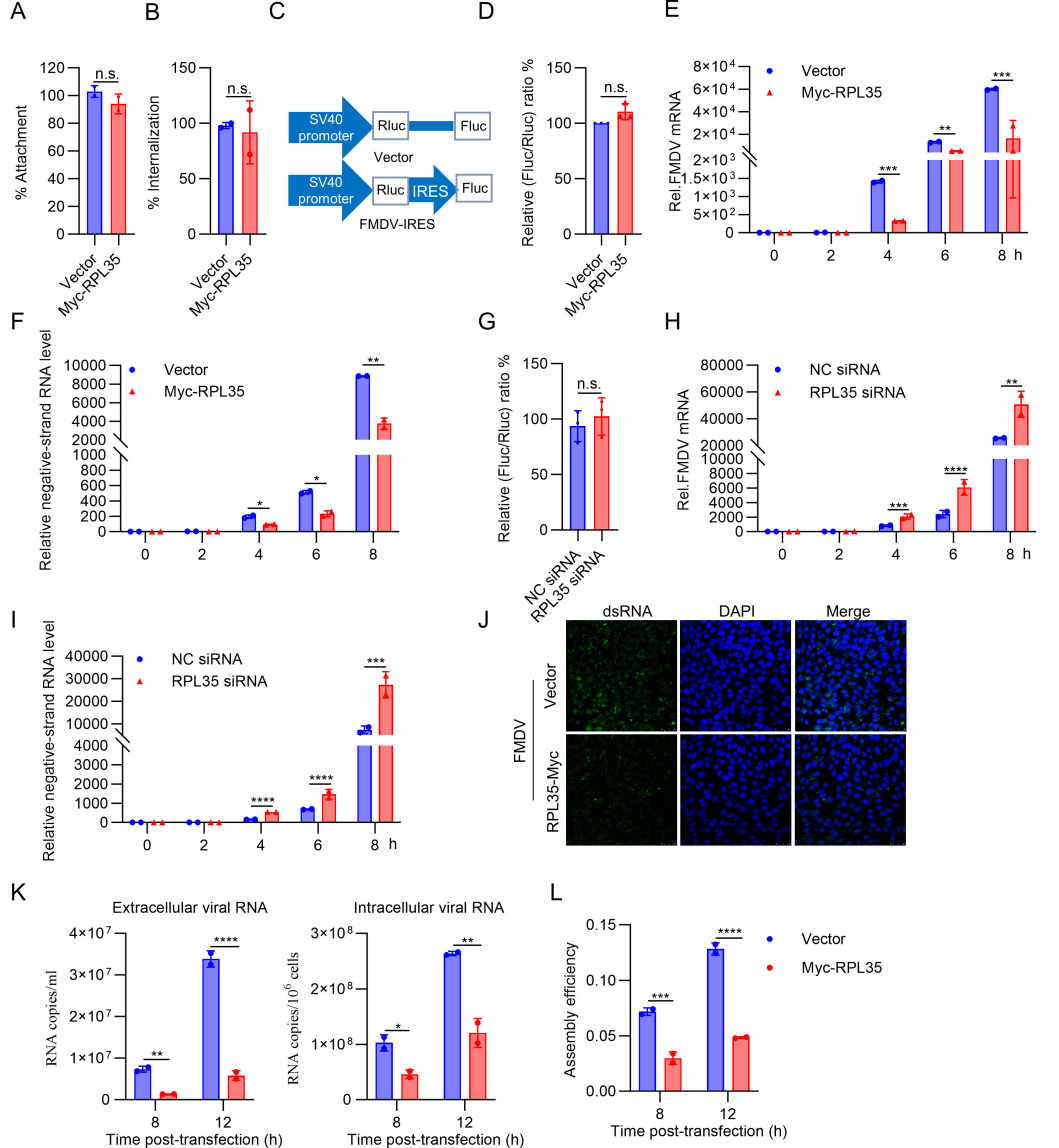
RPL35 affects FMDV RNA synthesis and virus assembly/release. (**A**) RPL35 did not affect the attachment of FMDV. PK-15 cells were transfected with either RPL35 or vector plasmids, followed by infection with FMDV at an MOI of 10 and incubation at 4°C for 1 h. After adsorption, unbound viruses were thoroughly washed away with ice-cold PBS. Cell-bound FMDV virions were quantified using RT-PCR. (**B**) RPL35 did not influence the internalization of FMDV. PK-15 cells were transfected with RPL35 or vector plasmids, then infected with FMDV at an MOI of 10 and incubated at 4°C for 1 h. Unbound FMDV virions were washed away with ice-cold PBS, and the cells were subsequently shifted to 37°C for 1 h. After additional washes, internalized FMDV virions were quantified by RT-PCR. (**C**) Schematic illustration of the bicistronic FMDV IRES construct. (**D**) RPL35 does not impact FMDV IRES-driven translation. PK-15 cells were transfected with the bicistronic FMDV-IRES construct along with RPL35 or vector plasmids. At 36 h post-transfection, the activities of Renilla luciferase (Rluc) and Firefly luciferase (Fluc) were measured. (**E and F**) Effect of RPL35 on viral RNA (vRNA) synthesis. PK-15 cells were transfected with RPL35 or vector plasmids, followed by infection with FMDV at an MOI of 1 at the indicated time points. Total (**E**) or negative-strand (**F**) viral RNA was quantified using RT-PCR. (**G**) Effect of endogenous RPL35 on FMDV IRES-driven translation. PK-15 cells were transfected with RPL35 siRNA or a negative control (NC) for 12 h and then transfected with bicistronic FMDV-IRES plasmids. At 36 h post-transfection, the activities of Renilla luciferase (Rluc) and Firefly luciferase (Fluc) were measured. (**H and I**) Effect of endogenous RPL35 on viral RNA (vRNA) synthesis. PK-15 cells were transfected with RPL35 siRNA or NC, followed by infection with FMDV at an MOI of 1 at the indicated time points. Total (**H**) or negative-strand (**I**) viral RNA was quantified using RT-PCR. (**J**) Overexpression of RPL35 significantly reduced the levels of FMDV double-stranded RNA (dsRNA). PK-15 cells were transfected with RPL35 or vector plasmids, then infected with FMDV at an MOI of 1 for 6 h. Anti-dsRNA antibodies were used to detect FMDV dsRNA synthesis via immunofluorescence assay (IFA). (**K and L**) Effect of RPL35 on the efficiency of FMDV virion assembly and release. PK-15 cells were transfected with RPL35 or vector plasmids, then infected with FMDV at an MOI of 0.5 for 8 and 12 h. Quantitative RT-PCR was employed to measure extracellular and intracellular viral RNA levels (**K**). The ratio of extracellular to intracellular viral RNA was calculated to assess virion assembly and release efficiency (**L**).*P < 0.05; **P < 0.01; ***P < 0.001; ****P < 0.0001; n.s., no statistical significance.

We next investigated which step of the viral replication cycle is targeted by RPL35. PK-15 cells were transfected with either RPL35 or control vector plasmids, followed by FMDV infection and incubation at 4°C for 1 h. After adsorption, unbound viruses were removed, and the abundance of cell-bound FMDV RNA was measured. No significant reduction in cell-bound FMDV RNA was observed in RPL35-expressing cells (Fig. 3A). To determine whether RPL35 affects FMDV internalization, the cells were inoculated with FMDV at 4°C for 1 h, followed by incubation at 37°C for an additional hour to allow internalization. RT-PCR analysis revealed that RPL35 had no impact on FMDV internalization (Fig. 3B). Based on these findings, we further explored whether RPL35 regulates viral mRNA translation or RNA synthesis. A bicistronic reporter plasmid was employed to assess FMDV IRES activity (Fig. 3C). In this system, translation of the first cistron (Renilla luciferase [Rluc]) is cap-dependent, whereas translation of the second cistron (firefly luciferase [Fluc]) is driven by FMDV IRES activity. Relative IRES activity was calculated as the ratio of Fluc to Rluc expression. The bicistronic reporter plasmid was transfected into RPL35-expressing or control cells, and cell lysates were collected 36 h post-transfection to measure Fluc and Rluc activities. The results showed no significant difference in FMDV IRES activity between RPL35-expressing and control cells (Fig. 3D). Additionally, we assessed the effect of endogenous RPL35 on FMDV translation and observed no impact (Fig. 3G).

During FMDV infection, the viral genomic RNA serves as a template for both translation and RNA replication, tightly coupling these processes. To evaluate the effect of RPL35 on viral RNA (vRNA) synthesis, total RNA and negative-strand RNA were quantified by RT-PCR. As shown in Fig. 3E and F, RPL35-expressing cells exhibited a significant reduction in the synthesis of viral negative-strand RNA compared to control cells. Conversely, knocking down RPL35 significantly promoted FMDV RNA synthesis (Fig. 3H and I). Additionally, RPL35-expressing and control cells were infected with FMDV for 6 h, and the synthesis of viral double-stranded RNA (dsRNA), a marker of FMDV replication, was detected by immunofluorescence. The results showed that overexpression of RPL35 significantly reduced the levels of FMDV dsRNA (Fig. 3J). Furthermore, we examined the effect of RPL35 on intracellular and extracellular viral RNA levels. The ratio of extracellular to intracellular RNA was used to assess the efficiency of virion assembly and release (28). At 8 and 12 h post-infection (hpi), RPL35-expressing cells exhibited reduced intracellular viral RNA levels compared to control cells, along with significantly decreased extracellular viral RNA levels (Fig. 3K). This resulted in a diminished extracellular-to-intracellular viral RNA ratio (Fig. 3L). These results suggest that RPL35 is involved in regulating FMDV virion assembly and release. In summary, these findings demonstrate that RPL35 plays dual roles in inhibiting FMDV replication and modulating virion assembly/release.

### RPL35 induces FMDV VP2 protein degradation in a proteasome-dependent manner

**Fig 4:**
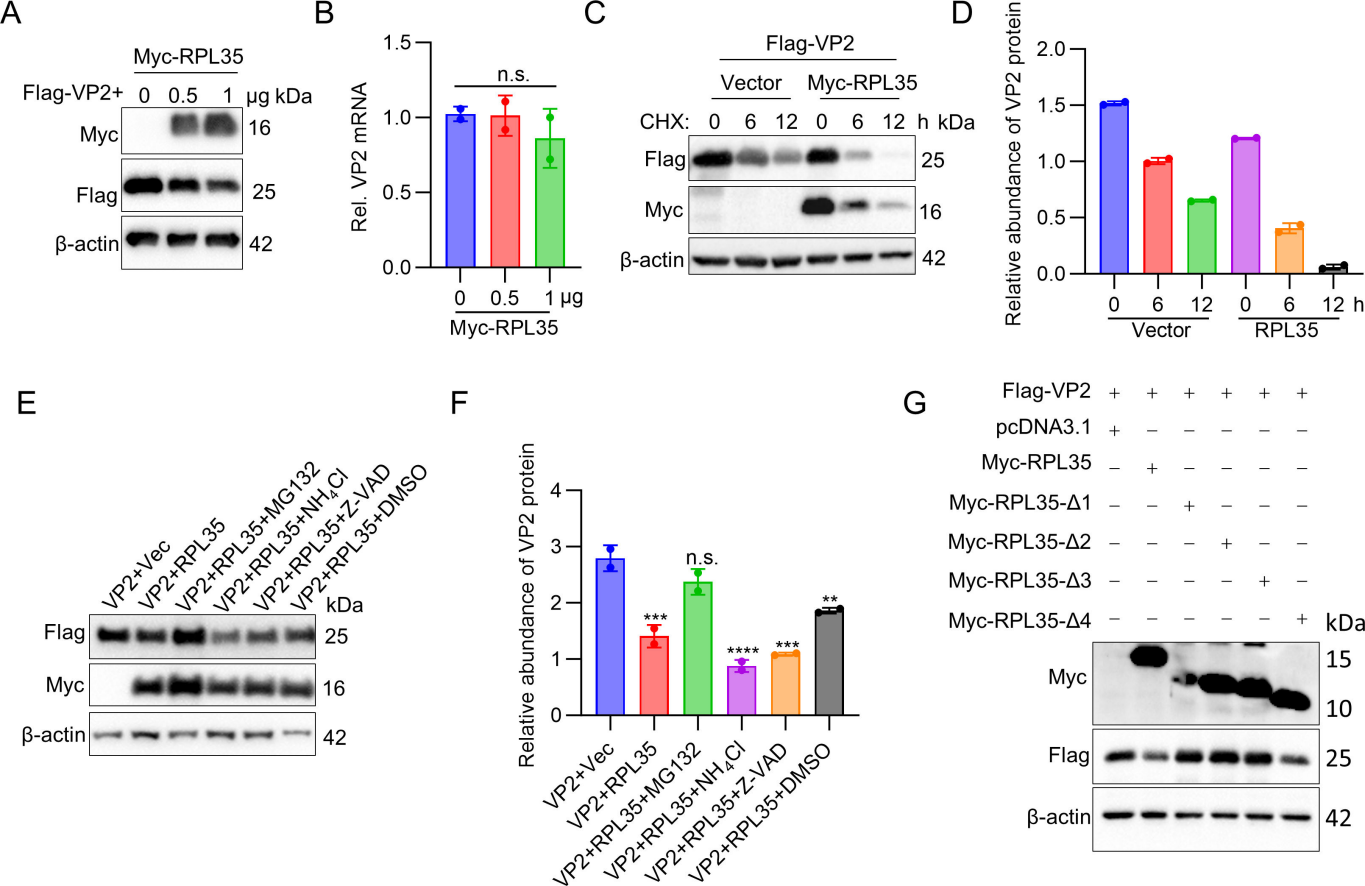
RPL35 induces FMDV VP2 protein degradation in a proteasome-dependent manner. (**A**) RPL35 induces a dose-dependent reduction of VP2. HEK293T cells were transfected with the Flag-VP2 plasmid along with increasing amounts of Myc-RPL35 plasmids. The expression levels of Flag-VP2 and Myc-RPL35 were assessed by immunoblotting. (**B**) Overexpression of RPL35 did not result in a significant decrease in VP2 mRNA levels. HEK293T cells were transfected with the VP2 plasmid and varying amounts of Myc-RPL35 plasmids. VP2 mRNA expression was analyzed by RT-PCR. (**C**) RPL35 regulates the half-life of the VP2 protein. HEK293T cells were co-transfected with Flag-VP2 and Myc-RPL35 plasmids. After 16 h, the cells were treated with cycloheximide (CHX) at 100 µg/mL for 0, 6, and 12 h prior to immunoblotting. (**D**) Relative fold change in VP2 abundance in panel C was determined by densitometric analysis. (**E**) RPL35 promotes the proteasomal degradation of VP2. Flag-VP2 was co-transfected with Myc-RPL35 in HEK293T cells, which were subsequently treated with DMSO (negative control), MG132 (20 µM), NH4Cl (20 mM), or Z-VAD-FMK (50 µM). (**F**) Relative fold change in VP2 abundance in panel E was determined by densitometric analysis. (**G**) The functional domain of RPL35 responsible for VP2 degradation was identified. HEK293T cells were transfected with Flag-VP2 and Myc-RPL35 or its truncated variants, and expression was analyzed by immunoblotting. **P < 0.01; ***P < 0.001; ****P < 0.0001; n.s., no statistical significance.

In the experimental process described above, we consistently observed that overexpression of RPL35 led to a decrease in VP2 protein levels. This observation prompted us to hypothesize that RPL35 may regulate VP2 protein stability or inhibit its expression. To test this hypothesis, we co-transfected Myc-RPL35 and Flag-VP2 into HEK293T cells. Our results indicated that VP2 protein levels decreased in a dose-dependent manner with RPL35 overexpression (Fig. 4A), whereas VP2 mRNA levels remained unchanged (Fig. 4B). To further investigate the suppressive effect of RPL35 on VP2 expression, we co-transfected HEK293T cells with either RPL35 or control vector plasmids alongside VP2 plasmids. The transfected cells were then treated with cycloheximide (CHX), a specific inhibitor of protein synthesis, to evaluate the half-life of VP2. Immunoblot analysis revealed that RPL35 overexpression significantly accelerated VP2 degradation, indicating that RPL35 regulates the half-life of VP2 (Fig. 4C and D).

To investigate the underlying mechanisms of RPL35-induced VP2 reduction, we examined the potential involvement of proteasomal, lysosomal, and caspase-dependent pathways. Myc-RPL35 and Flag-VP2 plasmids were co-transfected into HEK293T cells, which were subsequently treated with the proteasome inhibitor MG132, the lysosome inhibitor NH_4_Cl, or the pan-caspase inhibitor Z-VAD-FMK. As shown in Fig. 4E and F, treatment with MG132 restored VP2 levels in RPL35-overexpressing cells, whereas NH_4_Cl and Z-VAD-FMK had no comparable effect. These results suggest that RPL35 promotes VP2 degradation via the proteasome pathway.

To identify the functional domain of RPL35 responsible for VP2 degradation, Flag-VP2 was co-transfected with plasmids expressing various truncated forms of RPL35, and VP2 levels were subsequently assessed. The results demonstrated that the first, second, and third truncated mutants of RPL35 are all essential for VP2 degradation, as the absence of any of these regions abolished the degradation effect (Fig. 4G). In summary, these findings indicate that RPL35 induces the degradation of FMDV VP2 via the proteasome pathway, with the first three regions of RPL35 playing a critical role in this process.

### RPL35 catalyzes the K48-linked polyubiquitination of VP2

**Fig 5:**
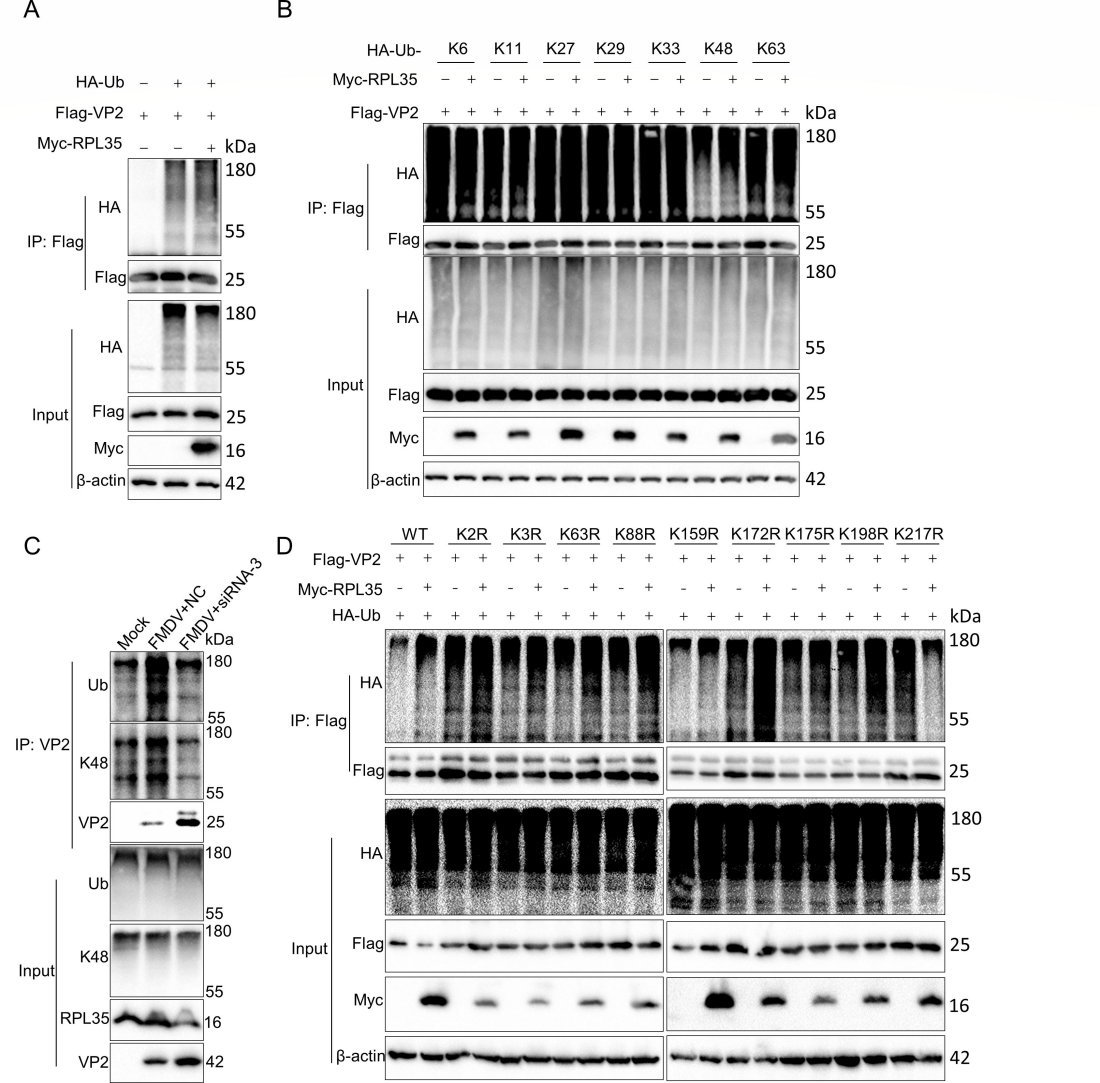
RPL35 catalyzes the K48-linked polyubiquitination of VP2. (**A**) RPL35 enhances the ubiquitination of VP2. Flag-VP2 and Myc-RPL35 were co-transfected into HEK293T cells along with HA-Ub, followed by treatment with MG132 (20 µM). VP2 was immunoprecipitated using a Flag antibody, and its ubiquitination was assessed with an HA antibody. (**B**) RPL35 facilitates K48-linked polyubiquitination of VP2. HEK293T cells were co-transfected with Myc-RPL35, Flag-VP2, and either wild-type (WT) or mutant forms of HA-Ub (K6, K11, K27, K29, K33, K48, or K63), then treated with MG132 (20 µM). VP2 was immunoprecipitated using a Flag antibody, and its ubiquitination was detected with an HA antibody. (**C**) Co-immunoprecipitation analysis of endogenous VP2 ubiquitination. PK-15 cells were infected with or without FMDV (MOI = 5) for 12 h. VP2 was immunoprecipitated, and its ubiquitination was detected using ubiquitin (Ub) and K48-specific antibodies. (**D**) Immunoblot analysis of ubiquitination of wild-type and mutant VP2 in HEK293T cells. Myc-RPL35, HA-K48-Ub, and Flag-VP2 or its mutants were co-transfected into HEK293T cells and treated with MG132 (20 µM). VP2 or its mutants were immunoprecipitated using a Flag antibody, and their ubiquitination was detected with an HA antibody.

Protein ubiquitination plays a crucial role in the proteasome-mediated degradation pathway (29, 30). To investigate the polyubiquitination of VP2, we overexpressed RPL35 or control vectors and treated the cells with MG132. As shown in Fig. 5A, MG132 treatment resulted in the accumulation of polyubiquitinated VP2 in cells overexpressing RPL35. To further characterize the types of VP2 polyubiquitination influenced by RPL35, we utilized a panel of ubiquitin (Ub) mutants (K6, K11, K27, K29, K33, K48, and K63). The results revealed that RPL35 specifically enhanced K48-linked polyubiquitination of VP2 (Fig. 5B). Additionally, experiments on endogenous ubiquitination demonstrated that RPL35 promoted both total ubiquitination and K48-linked ubiquitination of endogenous VP2 during viral infection (Fig. 5C). E3 ubiquitin ligases primarily mediate the ubiquitination of target proteins by modifying lysine residues (31). FMDV VP2 contains nine lysine residues distributed across its domains. To identify the specific ubiquitination sites on VP2, we generated a series of VP2 mutants (K2R, K3R, K63R, K88R, K159R, K172R, K175R, K198R, and K217R), in which lysine residues were replaced with arginine. We co-transfected HEK293T cells with Ub, RPL35, and either wild-type VP2 or its mutants, followed by MG132 treatment. Cell lysates were subjected to immunoprecipitation using anti-Flag antibodies. The results indicated that the K217R mutation significantly reduced the ubiquitination of VP2 mediated by RPL35 (Fig. 5D). In summary, these findings demonstrate that RPL35 catalyzes K48-linked polyubiquitination of VP2, with K217 identified as a critical ubiquitination site.

### The VP2 K217 residue is essential for RPL35-mediated restriction of virus replication

**Fig 6:**
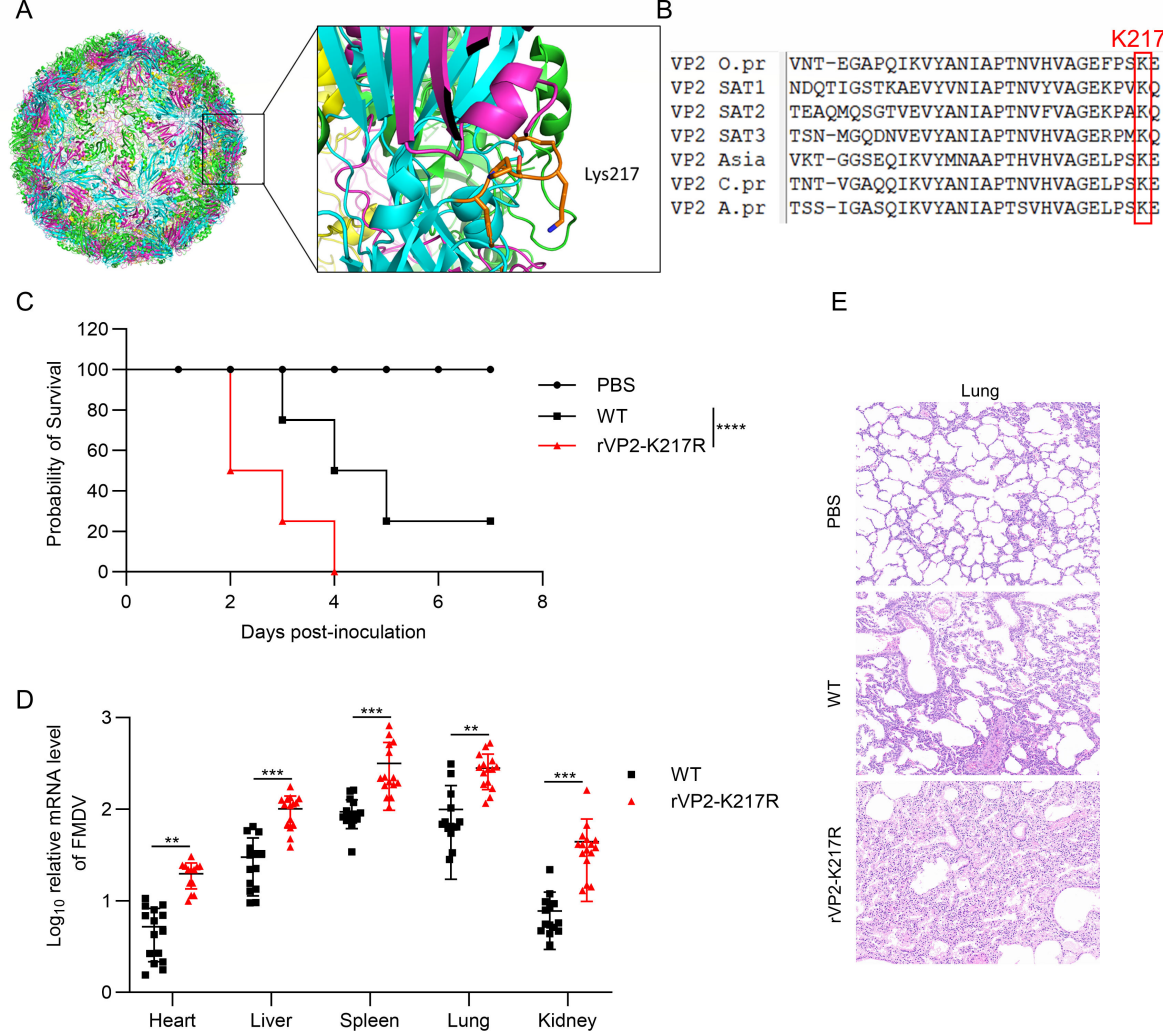
The VP2 K217 residue is essential for RPL35-mediated restriction of virus replication. (**A**) Lys217 is located on the surface of the virion. The PyMOL visualization was generated using the FMDV 3D structure from the Protein Data Bank (PDB ID: 7ENP). (**B**) Alignment of VP2 sequences from one representative of each FMDV serotype: Type O (GenBank accession no. JN998085.1), Type SAT1 (GenBank accession no. EF134950.1), Type SAT2 (GenBank accession no. EF134951.1), Type SAT3 (GenBank accession no. KR108964.1), Type Asia I (GenBank accession no. DQ989323.1), Type C (GenBank accession no. AJ133358.1), and Type A (GenBank accession no. MZ493234.1). (**C**) The survival rate of suckling mice was monitored daily for 7 days post-infection. Three-day-old suckling mice (*n* = 8 per group) were inoculated via cervicodorsal injection with PBS, wild-type (WT), or rVP2-K217R (20 LD_50_) in PBS, respectively. (**D**) FMDV mRNA levels were measured in the heart, liver, spleen, lung, and kidney of suckling mice challenged with either WT or rVP2-K217R (*n* = 5 per group). Data were obtained from three independent experiments. (**E**) Representative images of hematoxylin and eosin-stained lung sections from suckling mice (3 days post-infection) are shown. Scale bars represent 50 µm.**P < 0.01; ***P < 0.001; ****P < 0.0001.

The PyMOL illustration indicates that VP2 Lys217 is located on the surface of the virion, suggesting its potential role in binding to RPL35 (Fig. 6A). Subsequently, we compared the amino acid sequences of seven FMDV VP2 subtypes and found that the residues at position 217 (Lys) are conserved (Fig. 6B). Mutant viruses containing the K217R substitution (rVP2–K217R) were generated using reverse genetics technology, with the wild-type virus (WT) serving as a control to investigate the impact of Lys217 on FMDV replication. Suckling mice assays were conducted to compare the pathogenicity of the two viruses *in vivo*. Both viruses were virulent in suckling mice but exhibited different lethality levels. After 7 days post-inoculation, the WT group had a 25% survival rate, whereas all rVP2-K217R-infected mice died within 4 days (Fig. 6C). Additionally, tissue samples from the heart, liver, spleen, lung, and kidney of suckling mice at 3 days post-infection were analyzed by RT-PCR. The results revealed elevated levels of FMDV mRNA in tissues infected with rVP2-K217R compared with those infected with the wild-type virus (Fig. 6D). Furthermore, histopathological examination of the lungs showed that rVP2-K217R exacerbated lung lesions, such as alveolar shrinkage (Fig. 6E). These findings suggest that disruption of the Lys217 ubiquitination site in VP2 enhances FMDV pathogenesis *in vivo*.

### RPL35 recruits E3 ligase AMFR to degrade VP2 protein

**Fig 7:**
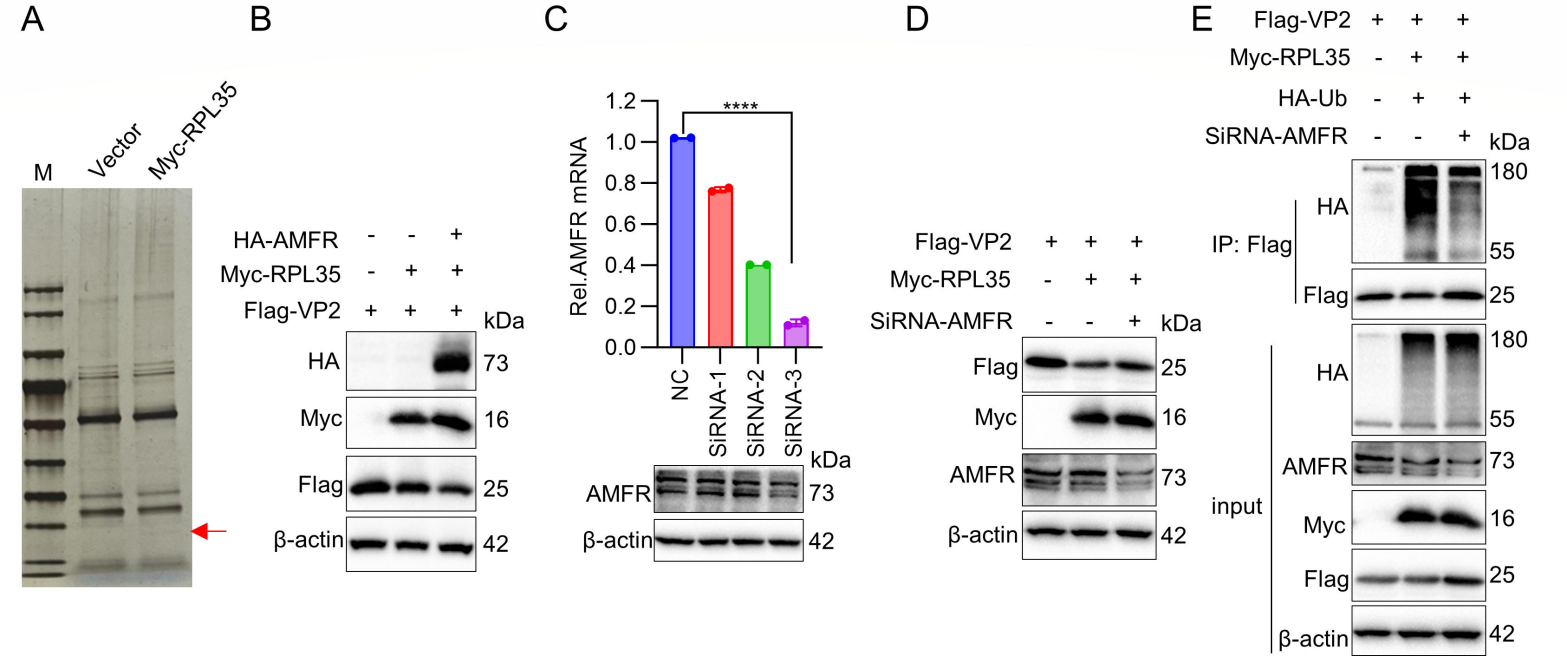
RPL35 recruits E3 ligase AMFR to facilitate VP2 protein degradation. (**A**) SDS-PAGE analysis of immunoprecipitation-purified host factors from PK-15 cells interacting with RPL35 protein. PK-15 cells were transfected with plasmids expressing either a control vector or Myc-RPL35. Cell lysates were immunoprecipitated using anti-Myc antibodies, resolved by SDS-PAGE, and subsequently stained with silver. (**B**) The effect of AMFR E3 ligase overexpression on RPL35-mediated degradation of VP2 was assessed. Immunoblot analysis was performed on PK-15 cells transfected with VP2, RPL35, or AMFR plasmids, using anti-HA, anti-Myc, and anti-Flag antibodies. (**C**) The efficiency of NC or AMFR siRNA in silencing AMFR expression was evaluated. PK-15 cells seeded in 6-well plates were transfected with 150 nM of either NC or AMFR siRNA (si-1, si-2, and si-3) for 36 h. Knockdown efficiency was assessed by RT-PCR (top) and immunoblot analysis (bottom). (**D**) The effect of AMFR E3 ligase knockdown on RPL35-mediated degradation of VP2 was evaluated. Immunoblot analysis was performed on PK-15 cells transfected with VP2, RPL35, or AMFR siRNA, using anti-Flag, anti-Myc, and anti-AMFR antibodies. (**E**) The influence of AMFR knockdown on RPL35-mediated ubiquitination of VP2 was analyzed. Immunoblot analysis using anti-HA antibodies was performed on proteins immunoprecipitated with anti-Flag antibodies from lysates of PK-15 cells transfected for 24 h with various combinations. **** *P* < 0.0001.

Although RPL35 modulates the stability of the VP2 protein through post-translational regulation, its intrinsic lack of E3 ubiquitin ligase activity prompted us to investigate potential collaborating ubiquitination enzymes. To elucidate the molecular mechanism underlying RPL35-mediated VP2 degradation, we employed immunoprecipitation-mass spectrometry (IP-MS) analysis and identified AMFR, a known E3 ubiquitin ligase (32), as a novel interacting partner of RPL35 (Fig. 7A). Subsequent functional validation revealed that exogenous overexpression of AMFR significantly enhanced RPL35-mediated VP2 proteolysis (Fig. 7B). To establish the necessity of AMFR in this degradation pathway, we systematically evaluated three distinct AMFR-targeting siRNAs. Quantitative analysis identified siRNA-3 as demonstrating superior knockdown efficiency (Fig. 7C), which was subsequently employed in loss-of-function studies. Depletion of AMFR markedly attenuated RPL35-induced VP2 degradation (Fig. 7D), accompanied by a reduction in VP2 ubiquitination levels (Fig. 7E). These findings collectively establish AMFR as the critical E3 ubiquitin ligase recruited by RPL35 to orchestrate VP2 ubiquitination and subsequent proteasomal degradation.

### The degradation of KPNA3 by FMDV proteins impairs the nuclear translocation of RPL35

**Fig 8:**
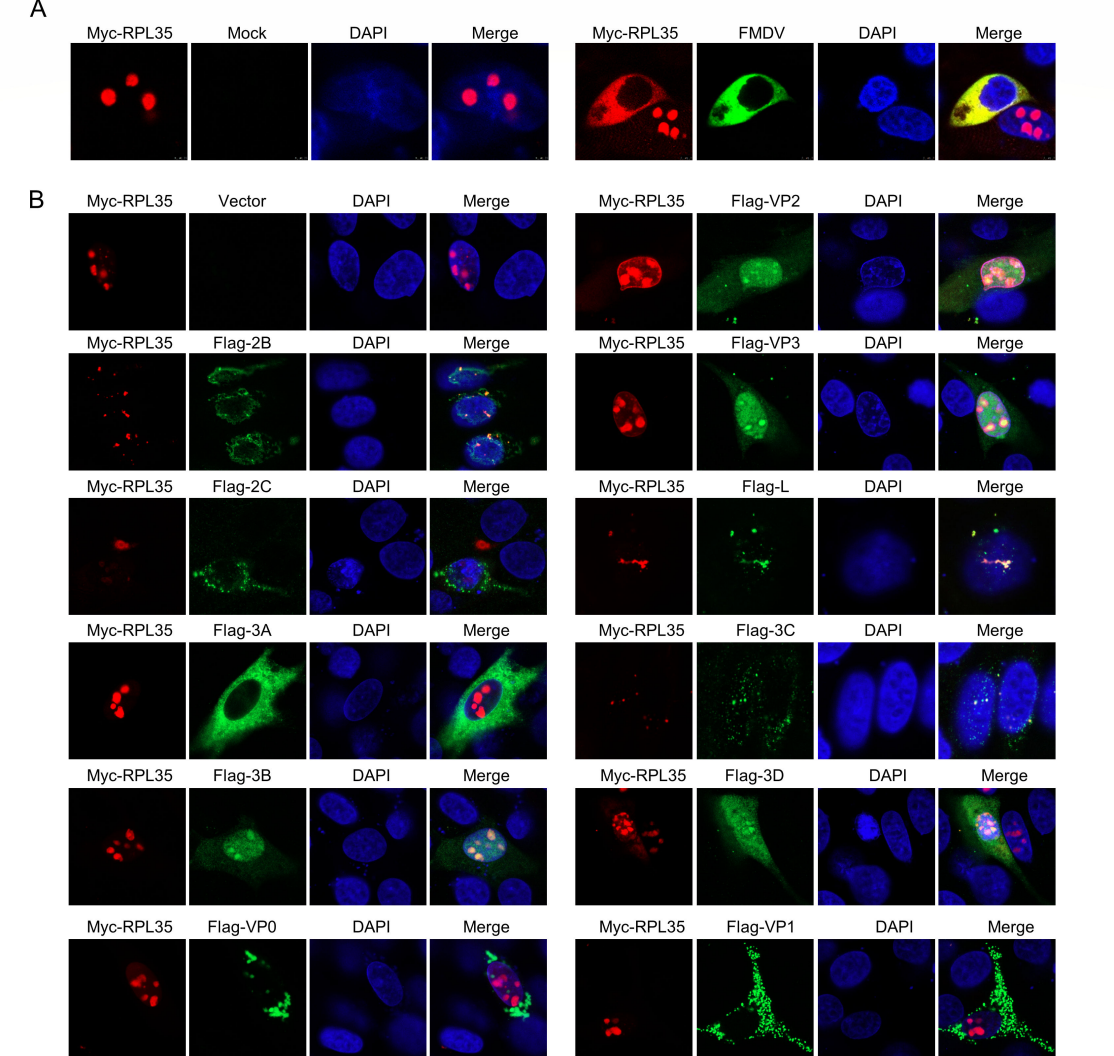
FMDV inhibits nuclear translocation of RPL35. (**A**) IBRS-2 cells were transfected with the Myc-RPL35 plasmids and subsequently either mock-infected or infected with FMDV at a multiplicity of infection (MOI) of 1. The cells were fixed at 8 h post-infection (hpi). After fixation, the cells were permeabilized and subjected to an immunofluorescence assay to analyze the localization of RPL35 (red) and FMDV (green). Nuclei were counterstained with 4',6-diamidino-2-phenylindole (DAPI) (blue). Imaging was performed using confocal microscopy. (**B**) IBRS-2 cells were transfected with RPL35 and various plasmids expressing FMDV structural and non-structural proteins. At 24 h post-transfection, the cells were fixed and analyzed for the localization of Flag-tagged viral proteins (green) and RPL35 (red) using an immunofluorescence assay (IFA). Nuclei were counterstained with DAPI (blue).

Normally, RPL35 mRNA is translated into protein in the cytoplasm. The protein then translocates to the nucleus (33), where it assembles with ribosomal RNA to form the 60S ribosomal subunit. This subunit, together with the 40S ribosomal subunit, exits the nucleus and reenters the cytoplasm. In the cytoplasm, the mature 60S and 40S subunits combine to form 80S ribosomes, which subsequently translate mRNA into protein (34–37). To investigate whether FMDV infection affects the nuclear translocation of RPL35, we performed confocal microscopy analysis. The results indicate that following FMDV infection, RPL35 does not enter the nucleus and remains in the cytoplasm (Fig. 8A). To further elucidate the viral factors that inhibit the nucleocytoplasmic translocation of RPL35, we examined the subcellular localization of RPL35 in cells ectopically expressing individual viral proteins using immunofluorescence. Our observations revealed that the FMDV proteins 2C, L, 2B, 3C, and 3D significantly inhibit the nuclear translocation of RPL35 (Fig. 8B).

**Fig 9:**
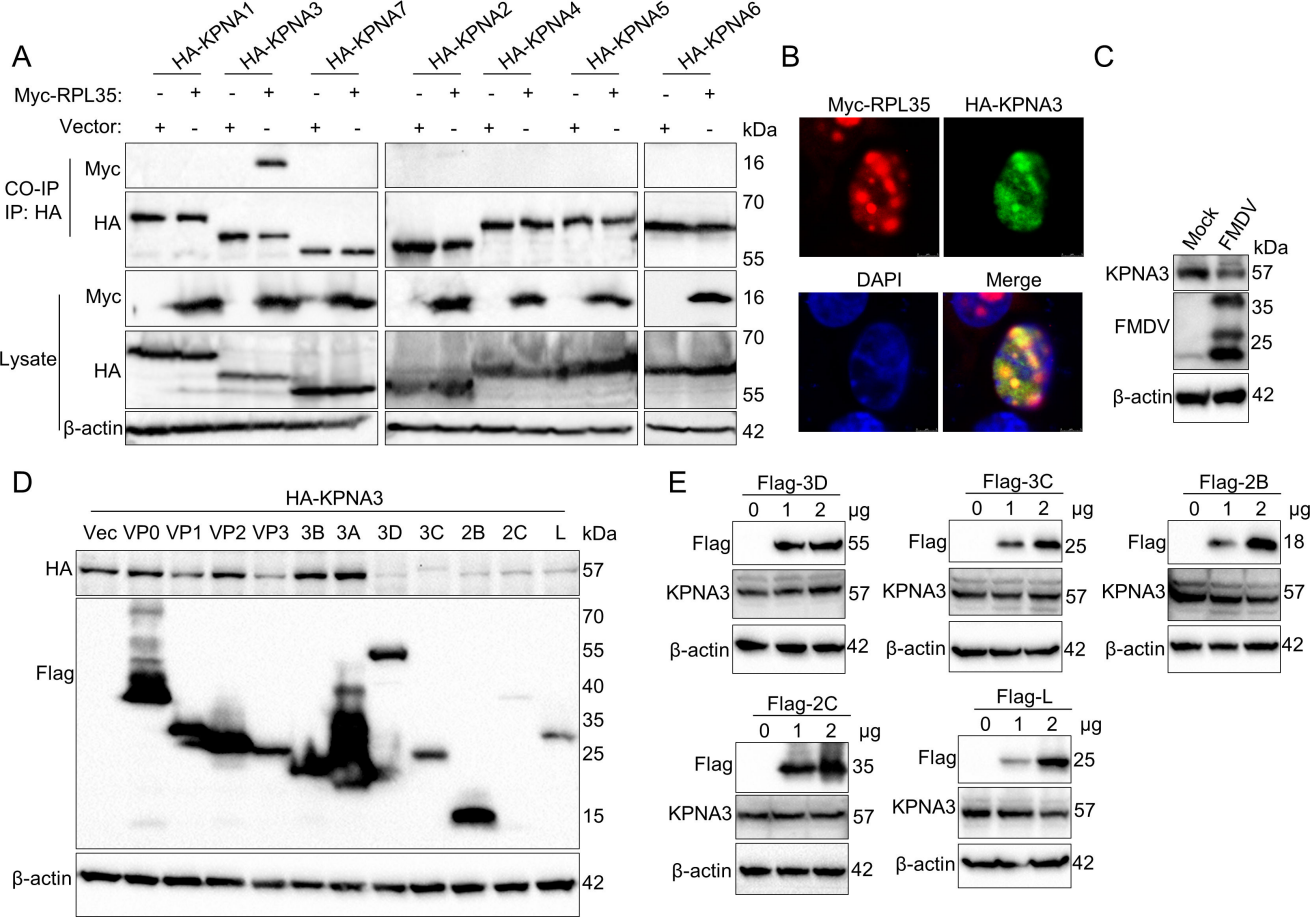
The degradation of KPNA3 by FMDV proteins impairs the nuclear translocation of RPL35. (**A**) Co-IP analysis was performed to investigate the interactions between RPL35 and KPNA proteins. IBRS-2 cells were co-transfected to express the indicated proteins. At 24 h post-transfection, cell lysates were prepared and analyzed by immunoblotting to assess input expression levels or subjected to Co-IP analysis. (**B**) Colocalization analysis of RPL35 and KPNA3 proteins was conducted using IBRS-2 cells transfected to express the indicated proteins. At 24 h post-transfection, the cells were fixed, stained with antibodies targeting the epitope tags, and examined by confocal microscopy. (**C**) Immunoblotting was used to detect the effect of FMDV infection on endogenous KPNA3 protein levels. IBRS-2 cells were infected with or without FMDV for 12 hours, and the results were analyzed accordingly. (**D**) The effect of viral proteins on KPNA3 was evaluated. HEK293T cells cultured in six-well plates were co-transfected with 1 µg of plasmids expressing Flag-tagged viral proteins and 1 µg of a plasmid expressing HA-KPNA3. At 24 h post-transfection, KPNA3 protein expression levels were assessed by western blot analysis. (**E**) The dose-dependent effect of virus proteins on endogenous KPNA3 was examined. Viral proteins 3D, 3C, 2B, 2C, and L were transfected into IBRS-2 cells at increasing doses, and endogenous KPNA3 and viral protein levels were detected after 24 h.

Nucleocytoplasmic transport is a vital process in eukaryotic cells (38). The molecular mechanisms underlying nuclear transport involve the nuclear transport receptor importin α, also known as karyopherin α (KPNA). KPNA comprises seven subtypes, specifically KPNA1 through KPNA7 (39, 40). To identify the primary subtype responsible for transporting RPL35 into the nucleus, we conducted immunoprecipitation experiments. The results indicate that RPL35 interacts with KPNA3 (Fig. 9A), and this interaction was confirmed by confocal microscopy (Fig. 9B). These findings suggest that KPNA3 is the transporter protein facilitating RPL35’s entry into the nucleus. This raises the possibility that FMDV may target nuclear localization signaling receptors for degradation. We examined whether KPNA3 was degraded in FMDV-infected cells. The results demonstrated that FMDV induces the degradation of endogenous KPNA3 (Fig. 9C). To identify which viral protein is responsible for KPNA3 degradation, HEK293T cells were co-transfected with KPNA3-expressing plasmids and plasmids expressing various Flag-tagged viral proteins. It was observed that the expression of the 3D, 3C, 2B, 2C, and L proteins significantly decreased KPNA3 abundance (Fig. 9D). However, dose-dependent analysis revealed that only the L and 2B proteins degraded KPNA3 in a dose-dependent manner (Fig. 9E). These results indicate that the FMDV proteins 2B and L induce KPNA3 degradation, thereby blocking RPL35 nuclear translocation.

## DISCUSSION

Ribosomal proteins (RPs), together with ribosomal RNA (rRNA), are essential components of ribosomes that facilitate the cellular process of protein biosynthesis, commonly known as "translation" (41, 42). Viruses, as small infectious agents with limited genomic capacity, must recruit various host factors, including RPs, to ensure their survival and replication (43). Recent research has increasingly elucidated the functional interplay between RPs and viral infections. Most of these interactions are critical for viral translation and replication, thereby enhancing viral infection and proliferation, whereas a smaller subset activates host cell defense mechanisms by triggering immune pathways against the virus (44). Exploring antiviral strategies based on RPs will guide future research. In this study, we identify the ribosomal protein RPL35 as a crucial host antiviral factor that targets the FMDV VP2 protein for proteasomal degradation. This discovery reveals a novel mechanism of host-mediated viral restriction and contributes to the development of potential therapeutic strategies against FMDV.

In contrast to established host-mediated ubiquitination pathways, such as those targeting influenza PB1 (e.g., TRIM32-mediated ubiquitination of PB1 [45]) or the rabies matrix protein (e.g., TRIM72-mediated ubiquitination of the matrix protein [46]), the RPL35-driven mechanism represents a unique antiviral strategy. Although both are host proteins that directly bind to viral proteins and induce K48-linked ubiquitination and degradation, RPL35 is not an E3 ligase. Instead, it operates through a distinct mechanism that recruits E3 ligases in host cells to ubiquitinate and degrade VP2. This highlights the diversity of host defense strategies against viral pathogens. Mechanistically, RPL35 overexpression reduces VP2 protein levels in a dose-dependent manner without affecting mRNA stability, whereas proteasome inhibition with MG132 fully restores VP2 accumulation. These findings position RPL35 as a post-translational regulator orchestrating viral protein turnover. Importantly, truncation analyses demonstrate that three distinct RPL35 domains cooperatively mediate VP2 degradation, suggesting a multivalent interaction mode that ensures robust antiviral activity. This contrasts with previous reports of single-domain antiviral ribosomal proteins, revealing an evolutionary refinement in host defense mechanisms.

Functional mapping has identified Lys217 of VP2 as the critical site for ubiquitination. Notably, the conservation of VP2’s Lys217 across various FMDV serotypes suggests the potential for developing pan-serotype ubiquitination enhancers, which could help overcome the limitations of current vaccines. The increased virulence observed in the rO-VP2K217R mutant indicates that FMDV may utilize residue-specific modifications to evade RPL35-mediated degradation, a strategy similar to those employed by other viruses to evade immune responses. For example, TRIM7 inhibits enterovirus replication and promotes the emergence of viral variants with increased pathogenicity (47). Additionally, TRIM21 binds to residue R95 of M1 and facilitates K48-linked ubiquitination of M1 at K242, leading to proteasome-dependent degradation and inhibition of H3, H5, and H9 IAV replication (48). Furthermore, the E3 ligase RNF5 interacts with VP1 and targets it for degradation through ubiquitination at Lys200, ultimately inhibiting FMDV replication (11).

Ribosomal proteins are typically involved in protein biosynthesis. However, recent studies have revealed an atypical role for ribosomal proteins in antiviral responses. This antiviral effect may extend beyond their traditional function in translation as ribosomal components. For example, ribosomal protein L9 interacts with the Gag protein of mouse mammary tumor virus (MMTV) to inhibit the assembly of MMTV virus particles (22). RPLP1 restricts HIV-1 transcription by disrupting C/EBPβ binding to the LTR (49). In this study, RPL35 exerts broad-spectrum antiviral effects by suppressing both viral RNA synthesis and virion assembly. As a crucial component of the 60S ribosomal subunit, RPL35 plays a significant role in protein translation and endoplasmic reticulum docking (50). The suppression of viral RNA synthesis mediated by RPL35 may result from infection-induced impairment of its nucleocytoplasmic trafficking, which disrupts the production of host proteins necessary for viral RNA replication, thereby indirectly attenuating viral RNA synthesis (33). This dual-phase inhibition—targeting both genomic replication and particle maturation—positions RPL35 as a gatekeeper at multiple stages of the viral lifecycle. Such multi-tiered antiviral activity may account for its potent suppression of viral titers, surpassing the efficacy of single-mechanism inhibitors. RPL35, as a ribosomal subunit component, plays a role independent of its translation function in antiviral responses. The aberrant localization of RPL35 induced by infection may selectively affect the translation of viral and host mRNAs; however, the mechanism underlying this selective effect requires further in-depth investigation.

Notably, proteomic screening has identified AMFR, an endoplasmic reticulum-associated E3 ubiquitin ligase, as a key mediator of RPL35-driven VP2 degradation. The recruitment of AMFR by RPL35 for the ubiquitination of VP2 reveals a novel function for AMFR that extends beyond its established role in STING activation (51). Although AMFR typically facilitates K27-linked ubiquitination of STING to enhance antiviral signaling (51), its repurposing here for K48-linked degradation of a viral structural protein broadens the functional repertoire of E3 ligases in antiviral immunity. Our findings align with emerging themes in host-pathogen interactions, where ubiquitination pathways act as double-edged swords–either promoting viral clearance or being exploited by viruses to undermine host defenses.

The dependence of FMDV on the stability of its structural protein VP2 for infectivity making VP2 proteins as a promising therapeutic target. Structural proteins are increasingly recognized as viable antiviral targets due to their conserved roles in viral assembly and entry. For example, pleconaril targets picornavirus capsids to block uncoating (52), whereas allosteric modulators of the hepatitis B virus core protein (CpAMs) disrupt capsid assembly (53, 54). Our research suggests that small molecules mimicking the interaction between RPL35 and VP2, or compounds that enhance AMFR-mediated ubiquitination, could destabilize VP2 and inhibit FMDV replication.

Furthermore, the identification of KPNA3 degradation as a viral countermeasure highlights the potential of stabilizing nuclear transport machinery to enhance host antiviral responses. For example, the African swine fever virus (ASFV) MGF360-12L disrupts KPNA3/4-dependent nuclear transport of NF-κB, thereby suppressing interferon responses (55). Similarly, the FMDV 3C protease degrades KPNA1 to inhibit STAT1/STAT2 nuclear translocation (56). Our study contributes to this paradigm by demonstrating that FMDV counteracts the antiviral function of RPL35 by inducing KPNA3 degradation, which restricts its nuclear trafficking. This reciprocal antagonism exemplifies the evolutionary arms race between host defenses and viral immune evasion.

**Fig 10:**
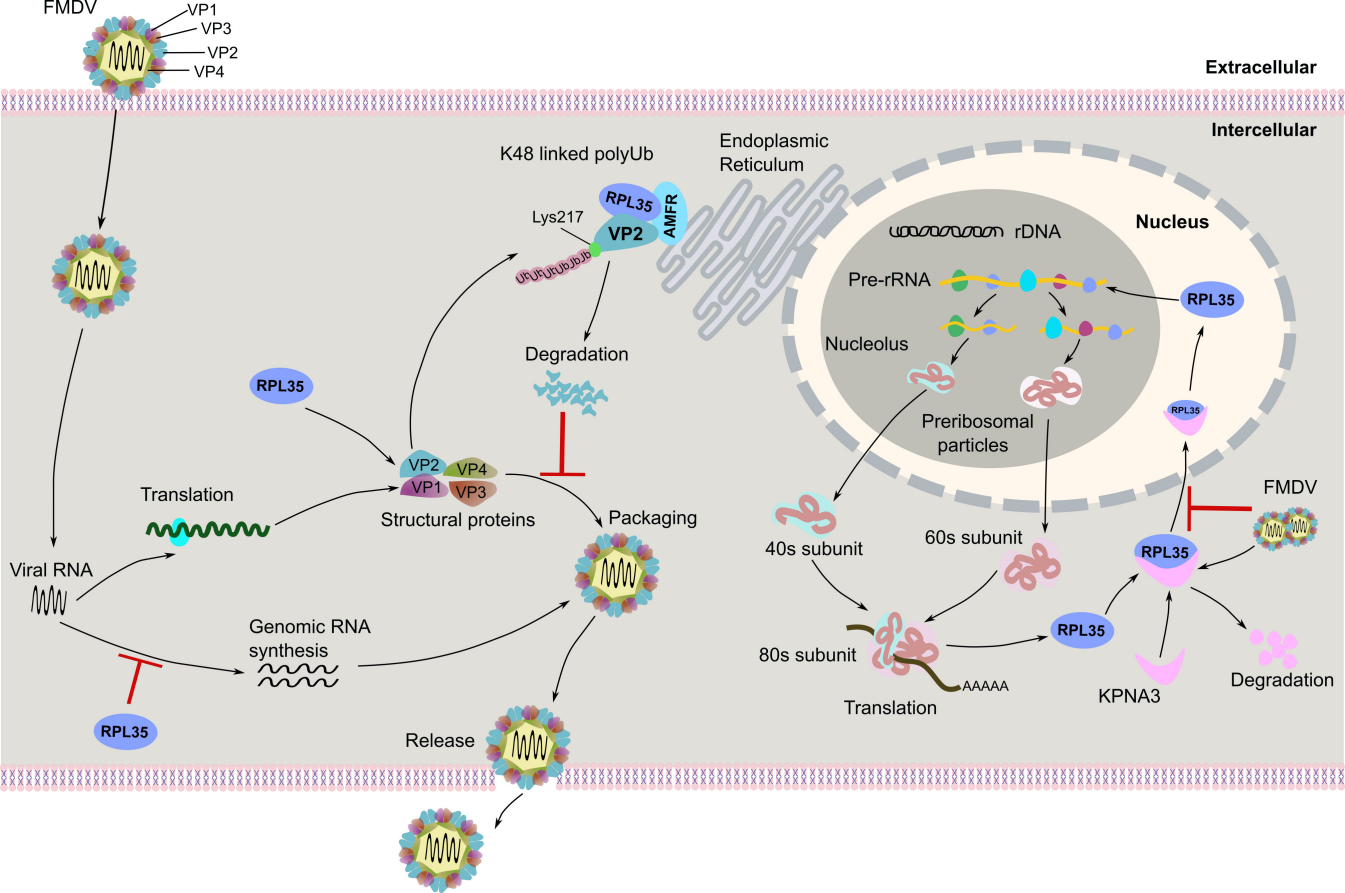
Schematic diagram of RPL35 inhibiting FMDV replication. In detail, RPL35 negatively regulates FMDV replication by recruiting AMFR, which facilitates the ubiquitination and subsequent degradation of VP2. Conversely, FMDV induces the degradation of KPNA3, thereby inhibiting the nuclear translocation of RPL35.

In conclusion, this study demonstrates that RPL35 negatively regulates FMDV replication by recruiting AMFR, which promotes the ubiquitination and degradation of VP2. Conversely, FMDV induces the degradation of KPNA3, thereby inhibiting RPL35’s nuclear translocation (Fig. 10). Our work connects virology fundamentals with drug discovery, offering a roadmap to combat an economically damaging veterinary pathogen.

## MATERIALS AND METHODS

### Cells and viruses

Porcine kidney cell lines (PK-15 and IBRS-2) and baby hamster kidney-21 (BHK-21) cells were cultured in minimum essential medium (MEM, Gibco, USA), whereas human embryonic kidney 293T (HEK293T) cells were maintained in Dulbecco’s modified Eagle medium (DMEM, Gibco, USA). All culture media were supplemented with 10% fetal bovine serum (FBS), 1% streptomycin (0.2 mg/mL), and penicillin (200 U/mL). Cells were incubated at 37°C in a humidified atmosphere containing 5% CO_2_. For viral infection experiments, the FMDV O/BY/CHA/2010 strain was used.

### Reagents and antibodies

MG132 was purchased from Merck & Co (Germany), whereas NH_4_Cl and benzyloxycarbonyl (Cbz)-Val-Ala-Asp (OMe)-fluoromethylketone (Z-VAD-FMK) were obtained from Sigma-Aldrich (USA). The following antibodies were used in this study: anti-HA (Biolegend, Cat #901513), anti-Flag (Sigma-Aldrich, Cat #F1804), anti-Myc (Sigma-Aldrich, Cat #M5546), anti-β-actin (Sigma-Aldrich, Cat #A5441), anti-RPL35 (Proteintech, Cat #14826-1-AP), anti-dsRNA (Sigma-Aldrich, Cat #MABE1134), anti-ubiquitin (Cell Signaling Technology (CST), Cat #3933), anti-K48-linkage specific polyubiquitin (CST, Cat #4289), and anti-K63-linkage specific polyubiquitin (D7A11) (CST, Cat #5621). Secondary antibodies included Mouse anti-goat IgG/Alexa Fluor 594 (Invitrogen, Cat #A11005), Rabbit anti-goat IgG/Alexa Fluor 488 (Invitrogen, Cat #A11008), Rabbit anti-goat IgG/Alexa Fluor 594 (Invitrogen, Cat #A11037), and Mouse anti-goat IgG/Alexa Fluor 488 (Invitrogen, Cat #A28175). Guinea pig anti-FMDV positive serum and mouse anti-VP2 antibody were provided by the Lanzhou Veterinary Research Institute (LVRI).

### Plasmids

The genes encoding VP2, RPL35, RPL35 (deletion of residues 1–30), RPL35 (deletion of residues 31–60), RPL35 (deletion of residues 61–90), RPL35 (deletion of residues 91–123), AMFR, and KPNA1-7 were cloned into the pcDNA3.1/myc-His A or pxj41 vector (Invitrogen, USA) to generate Flag-VP2, Myc-RPL35, Myc-RPL35-Δ1, Myc-RPL35-Δ2, Myc-RPL35-Δ3, Myc-RPL35-Δ4, HA-AMFR, and HA-KPNA1-7, respectively. Additionally, a series of Flag-tagged VP2 mutants (K2R, K3R, K63R, K88R, K159R, K172R, K175R, K198R, and K217R), in which lysine residues were substituted with arginine, were constructed using site-directed mutagenesis PCR. The pCMV-HA-Ub wild-type (WT) and its mutants (K6, K11, K27, K29, K33, K48, and K63) were obtained from BioZY Co., LTD. The primer pairs used for PCR amplification are listed in Table 1. All constructed plasmids were validated by DNA sequencing to ensure accuracy.

**TABLE 1:** PCR primer pairs used in this study

Primer	Sequence (5' to 3')^*a*^	Target gene
VP2-F	CGTCTA*GCTAGC*GATAAGAAAACCGAGGAGAC	FMDV VP2 gene
VP2-R	CGC*GGATCC*CTCTTTGGAAGGGAACTCAC	
RPL35-F	CGTCTA*GCTAGC*ATGGCCAAGATTAAGGCTCG	RPL35 gene
RPL35-R	CGC*GGATCC*GGCCTTGACGGCGAACTTCC	
KPNA1-F	CGTCTA*GCTAGC*ATGCTTCCAGAGAAAGACCG	KPNA1
KPNA1-R	CGC*GGATCC*AAGCTGGAACCCTTCCATAG	
KPNA2-F	CGTCTA*GCTAGC*ATGTCCACCAATGAGAATGCTA	KPNA2
KPNA2-R	CGC*GGATCC*AAAGTTGAAGGTCCCAGTAGC	
KPNA3-F	CGTCTA*GCTAGC*ATGGCCGAGAACCCCGGCTTGGA	KPNA3
KPNA3-R	CGC*GGATCC*AAAATTAAATTCTTTCGTTTGAAG	
KPNA4-F	CGTCTA*GCTAGC*ATGGCGGACAGCGAGAAACTGGACA	KPNA4
KPNA4-R	CGC*GGATCC*AAACTGGAACCCTTCTGTTGGT	
KPNA5-F	CGTCTA*GCTAGC*ATGAATGCCATGGCTAGTCCAGG	KPNA5
KPNA5-R	CGC*GGATCC*AAGGTGAAACCCTTCCATTG	
KPNA6-F	CGTCTA*GCTAGC*ATGTTAGAGACCATGGCGAGCCCAG	KPNA6
KPNA6-R	CGC*GGATCC*TAGCTGGAAGCCCTCCATGGGGGCC	
KPNA7-F	CGTCTA*GCTAGC*ATGCCGATTTTAGAAGCTCCCG	KPNA7
KPNA7-R	CGC*GGATCC*GGTTTTTGTTAAGGGCGTT	

^*a*^ The italics represent enzymes.

### Co-immunoprecipitation assay (Co-IP) and immunoblot analysis

HEK293T cells were cultured in 10-cm dishes until reaching monolayer confluency, followed by co-transfection with the indicated plasmids. After transfection, the cells were lysed in 1 mL of lysis buffer containing 20 mM Tris (pH 7.5), 150 mM NaCl, 1% Triton X-100, 1 mM EDTA, 10 µg/mL aprotinin, 10 µg/mL leupeptin, and 1 mM PMSF. The lysates were then incubated with 0.5 mg of the appropriate antibody and 40 µL of protein G-Sepharose (GE Healthcare) in 20% ethanol for 12 h at 4°C. The Sepharose beads were washed three times with 1 mL of lysis buffer supplemented with 500 mM NaCl. The immunoprecipitated proteins were subsequently analyzed by immunoblotting. For immunoblot analysis, the proteins were separated by SDS-PAGE and transferred onto an Immobilon-P membrane (Millipore, USA). The membrane was blocked and probed with the appropriate primary and secondary antibodies. Protein-antibody complexes were visualized using enhanced chemiluminescence (ECL) detection reagents (Thermo, USA).

### Immunofluorescence microscopy

Virus-infected or transfected cells were fixed with 4% paraformaldehyde for 30 min and permeabilized with 0.1% Triton X-100 for 15 min. After blocking with 5% bovine serum albumin (BSA) at 4°C for 4 h, the cells were incubated with the appropriate primary antibody, followed by staining with Alexa Fluor 488- or 594-conjugated secondary antibodies. Cellular images were acquired using a laser-scanning confocal microscope (LSCM, Leica SP8, Solms, Germany).

### Adsorption and internalization assay

In the FMDV adsorption assay, cells overexpressing RPL35 or the empty vector were incubated with FMDV at a multiplicity of infection (MOI) of 10 at 4°C for 1 h. Following adsorption, unbound viruses were removed by washing with ice-cold PBS, and the amount of cell-associated viral RNA was quantified by RT-PCR. For the FMDV internalization assay, cells were first incubated with FMDV at an MOI of 10 at 4°C for 1 h to allow viral attachment. After removing unbound viruses with ice-cold PBS, the cells were shifted to 37°C for 1 h to promote viral internalization. Non-internalized viruses were then eliminated by treatment with PBS containing proteinase K, and the levels of internalized viral RNA were measured using RT-PCR.

### TCID_50_ titration

BHK-21 cells were used to titrate the released infectious virus. The infected cells were harvested at the indicated time post-infection, and the titers were determined in terms of 50% tissue infection dose (TCID_50_)/100 μL by using the Reed-Muench method (57).

### Reverse genetics

The strategy for construction of the plasmid used to produce the recombinant virus was as described previously by our laboratory (58). At 48 h post-transfection, the cell supernatants were harvested by centrifugation at 6,000 × *g* for 10 min at 4°C and passaged in BHK-21 cells four times. The recovered viruses were named as rVP2-K217R mutant virus and WT virus, which were stored in the National Foot-and-Mouth Disease Reference Laboratory (ABSL-3), Lanzhou Veterinary Research Institute, Chinese Academy of Agricultural Sciences, following the standard protocols and biosafety regulations provided by the Institutional Biosafety Committee. Both viruses were amplified by PCR using the primers used previously, and the PCR products were sequenced using the amplified primers.

### RNA extraction and RT-PCR

Total RNA was isolated utilizing TRIzol Reagent (Invitrogen, USA), followed by cDNA synthesis from the extracted RNA samples employing M-MLV reverse transcriptase (Promega, USA) and random hexamer primers (TaKaRa, Japan). The resulting cDNA served as the template for assessing the expression levels of FMDV RNA and host cellular mRNA. RT-PCR was conducted using the Mx3005P QPCR system (Agilent Technologies, USA) and SYBR Premix ExTaq reagents (TaKaRa, Japan) to quantify RNA levels, with the specific primers detailed in Table 2. The glyceraldehyde-3-phosphate dehydrogenase (GAPDH) gene was utilized as an internal reference control. The relative mRNA expression was determined through the comparative cycle threshold (2^−ΔΔCT^) method.

**TABLE 2:** Primers used for mRNA quantification

Primer	Sequence (5' to 3')
GAPDH forward	ACATGGCCTCCAAGGAGTAAGA
GAPDH reverse	GATCGAGTTGGGGCTGTGACT
RPL35 forward	TGCTGAAACAACTGGAGGAC
RPL35 reverse	GAGGTTCTCTTTCTGGGTCT
FMDV forward	CACTGGTGACAGGCTAAGG
FMDV reverse	CCCTTCTCAGATTCCGAGT
VP2 forward	AGTTGTCCAGGCGGAACGGT
VP2 reverse	CTCATGTAGGCGTATGAGTC

### RNA interference (RNAi)

The small interfering RNA (siRNA) utilized in the RNA interference (RNAi) experiment was synthetically produced by GenePharma in Shanghai, China. The silencing of endogenous RPL35 was achieved through the transfection of RPL35 siRNA (5′-GAGCUGCUGAAACAACUGGTT-3′, 5′-GCGUUCUCACCGUCAUCAATT-3′, or 5′-GAGGAGAACCUGAAGACCATT-3′) into PK-15 cells using Polyplus jetPRIME transfection reagent. A non-targeting siRNA (NC siRNA: 5′-UUCUCCGAACGUGUCACGUTT-3′) served as a negative control in the study.

### Cell viability assay

The Cell Counting Kit-8 (CCK-8) from Yeasen was employed to evaluate cell viability in this study. Cells were plated in 96-well plates and incubated for 6–8 h before being treated with 10 μL of CCK-8 solution as per the manufacturer’s guidelines. Cell viability was assessed by measuring the absorbance of the cells at OD_450_ nm after a 2-h incubation period.

### Mouse infection

For survival analysis, 3-day-old suckling mice were subcutaneously inoculated in the neck with 0.2 mL of PBS, WT, or rVP2-K17R (20LD_50_) in PBS. The survival rates of all groups (*n* = 8 per group) were monitored for a period of 7 days. For the histopathological analysis, three mice per group were euthanized on day 3 post-infection to check for lesions in the lungs. For RT-PCR analysis, five mice per group were euthanized on day 3 post-infection to check for virus replication in the muscle, heart, liver, spleen, lung, kidney, and duodenum.

### Histological assessment

Following euthanasia of the mice, their tissues were gathered and promptly preserved in 10% neutral-buffered formalin. The preserved tissues were then embedded in paraffin, sectioned, and subjected to staining with hematoxylin and eosin (H&E) for subsequent histopathological examinations, as conducted by Wuhan Servicebio Technology Co., Ltd.

### Statistical analysis

All data were presented as means ± SD and analyzed using GraphPad Prism software (version 10.1.0). Individual statistical tests are specified within the figure legends. For data with two groups, unpaired Student’s *t* tests were used under the assumption of normality. Data with more than two groups were analyzed by analysis of variance (ANOVA) under the assumption of normality. In general, at least three independent biological replicates (*n*) were carried out for each experiment. Data were reproduced in independent experiments as indicated in the legends. Significant differences are denoted in the figures as follows: *P < 0.05; **P < 0.01; ***P < 0.001; ****P < 0.0001; n.s., no statistical significance.

## Data Availability

The data to support the findings of this study are included within the article.
